# Supplementary material for: A-to-I RNA co-editing predicts clinical outcomes and is associated with immune cells infiltration in hepatocellular carcinoma
Source: Commun Biol. 2024 Jul 9;7:838. doi: 10.1038/s42003-024-06520-y (PMC11233613; doi:10.1038/s42003-024-06520-y)
Supplement: Supplementary file 2 — Supplementary Information [file 42003_2024_6520_MOESM2_ESM.pdf]

Supplementary Information

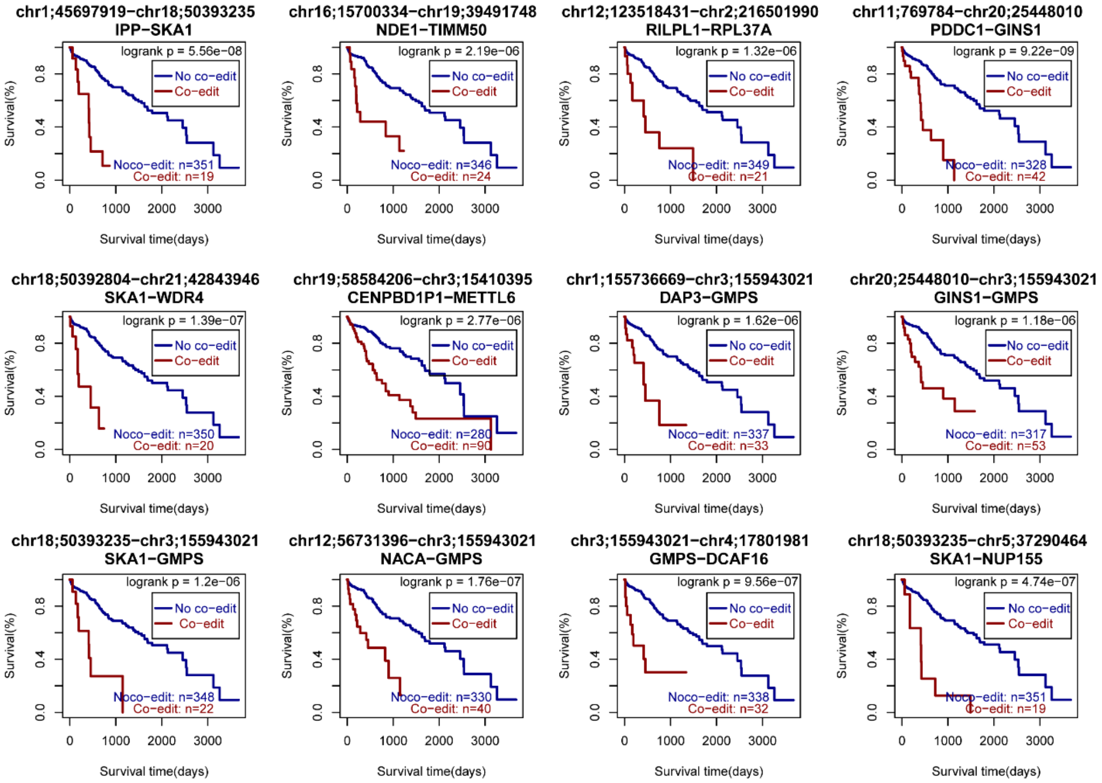

Supplementary Fig. 1. The prognostic related RNA co-editing pairs without adjusting the effect of single RNA editing sites.

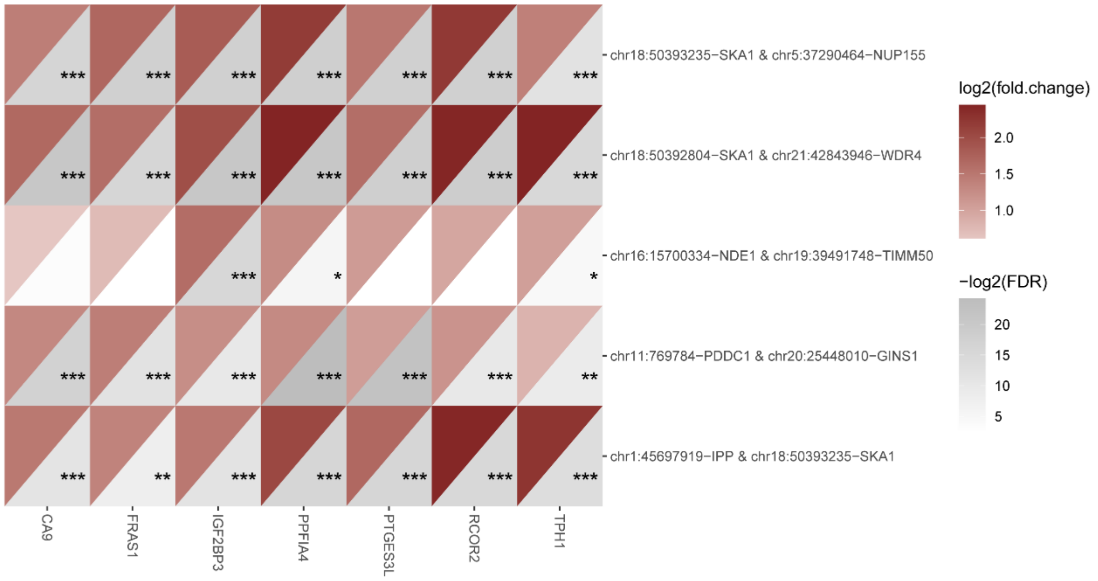

Supplementary Fig. 2. The expression levels of these seven genes were significantly elevated in RNA co-editing samples compared to those without co-editing events. Two-sided Wilcoxon–Mann–Whitney test. * FDR < 0.05, ** FDR < 0.01, * FDR < 0.001.**

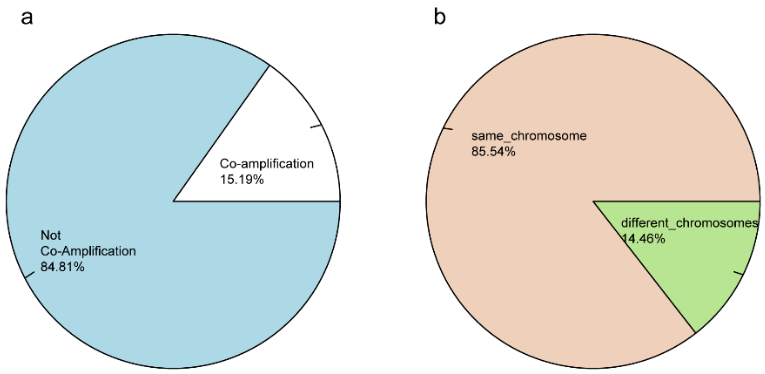

Supplementary Fig. 3. The percentage of RNA co-editing pairs exhibited co-amplification. a. Total RNA co-editing pairs. b. The percentage of co-amplificated RNA co-editing pairs located on the same chromosome.

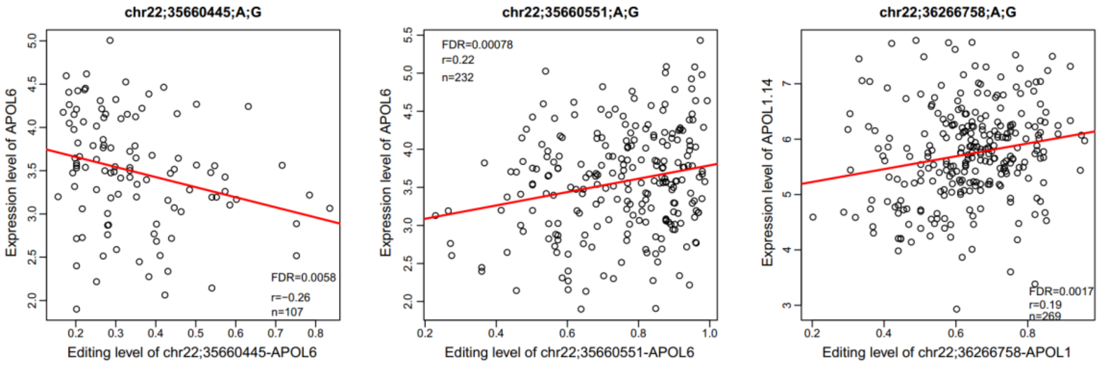

Supplementary Fig. 4. Three editing sites within interferon-related genes whose editing levels were significantly correlated with the genes' expression levels (Pearson correlation, Benjamini-Hochberg adjusted FDR < 0.05).

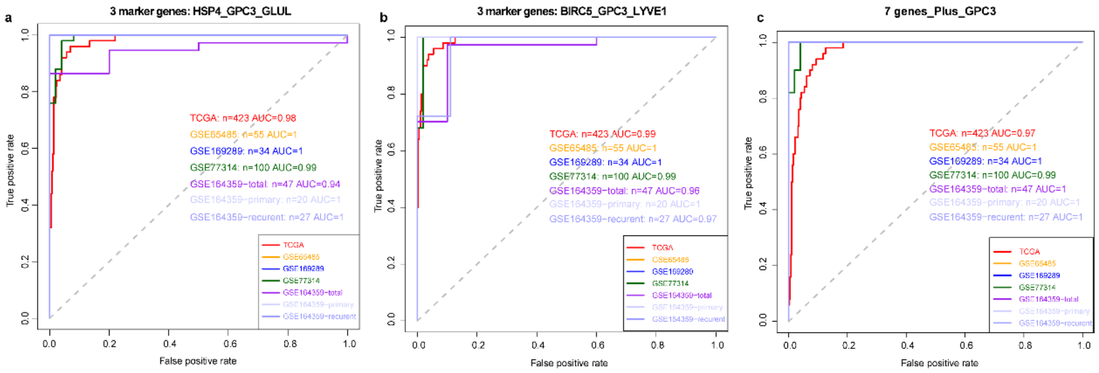

Supplementary Fig. 5. ROC curves of other diagnostic maker genes to predict sample status in TCGA and four independent GEO cohorts. a. ROC curves of 3-gene signature consisting of *HSP70*, *GPC3* and *GLUL*. b. ROC curves of 3-gene signature comprising *GPC3*, *LYVE1* and *BIRC5*. c. ROC curves of seven genes combined with *GPC3*.

Supplementary Table 1: The genomic annotation of 5 prognostic RNA co-editing pairs.

RNA editing1	RNA editing2	Gene 1(editing 1)	Gene 2(editing 2)	Annotation (editing1)	Annotation (editing2)
chr1;45697919	chr18;50393235	IPP	SKA1	intronic	3’UTR
chr16;15700334	chr19;39491748	NDE1	TIMM50	intronic	3’UTR
chr11;769784	chr20;25448010	PDDC1	GINS1	3’UTR	3’UTR
chr18;50392804	chr21;42843946	SKA1	WDR4	3’UTR	intronic
chr18;50393235	chr5;37290464	SKA1	NUP155	3’UTR	intergenic

Supplementary Table 2: The results of multivariate survival analysis of 5 prognostic RNA co-editing pairs.

**chr1;45697919 & chr18;50393235**		**HR(95% CI)**	**p-value**
**Co-editing**			
	No	1(reference)	
	Yes	3.11(1.19-8.14)	**0.02 ***
**Gender**			
	Female	1(reference)	
	Male	0.79(0.47-1.31)	0.35
**Ages**		1.00	**0.01***
**Stage**			
	T1	1(reference)	
	T2	1.22(0.64-2.36)	0.55
	T3	1.65(0.88-3.09)	0.12
	T4	2.76 (0.92-8.31)	0.07
**Grade**			
	Grade G1	1(reference)	
	Grade G2	1.66(0.64-4.35)	0.30
	Grade G3	2.20(0.81-5.95)	0.12
	Grade G4	3.81(0.95-15.20)	0.06
**Fetoprotein**		1.00(1.00-1.00)	0.35
**chr16;15700334 & chr19;39491748**		**HR(95% CI)**	**p-value**
**Co-editing**			
	No	1(reference)	
	Yes	3.60(1.67-7.78)	**0.001 ****
**Gender**			
	Female	1(reference)	
	Male	0.90(0.54-1.50)	0.69
**Ages**		1.00	**0.002****
**Stage**			
	T1	1(reference)	
	T2	1.13(0.58-2.18)	0.73
	T3	1.49(0.79-2.81)	0.21

T4	4.48(1.70-11.79)	**0.002 ****
**Grade**		
Grade G1	1(reference)	
Grade G2	1.55(0.59-4.07)	0.38
Grade G3	2.31(0.86-6.23)	0.10
Grade G4	5.74(1.57-21.01)	**0.008****
**Fetoprotein**	1.00(1.00-1.00)	0.32
**chr11;769784 & chr20;25448010**	**HR(95% CI)**	**p-value**
**Co-editing**		
No	1(reference)	
Yes	5.68(2.75-11.73)	**2.8e-6 *****
**Gender**		
Female	1(reference)	
Male	1.05(0.61-1.80)	0.85
**Ages**	1.00	**0.003****
**Stage**		
T1	1(reference)	
T2	1.22(0.63-2.38)	0.55
T3	1.89(0.99-3.61)	**0.05 ***
T4	4.46(1.69-11.76)	**0.003 ****
**Grade**		
Grade G1	1(reference)	
Grade G2	1.68(0.64-4.43)	0.30
Grade G3	2.12(0.78-5.82)	0.14
Grade G4	4.17(1.10-15.86)	**0.04 ***
**Fetoprotein**	1.00(1.00-1.00)	0.41
**chr18;50392804 & chr21;42843946**	**HR(95% CI)**	**p-value**
**Co-editing**		
No	1(reference)	
Yes	7.64(2.96-19.73)	**2.7e-5 *****
**Gender**		
Female	1(reference)	
Male	0.92(0.55-1.53)	0.74
**Ages**	1.00	**0.005****

**Stage**			
	T1	1(reference)	
	T2	1.28(0.66-2.47)	0.46
	T3	1.60(0.85-3.00)	0.14
	T4	5.40(2.03-14.33)	**0.0007 *****
**Grade**			
	Grade G1	1(reference)	
	Grade G2	1.62(0.62-4.26)	0.33
	Grade G3	2.09(0.77-5.67)	0.15
	Grade G4	5.29(1.43-19.62)	**0.01 ***
**Fetoprotein**		1.00(1.00-1.00)	0.20
**chr18;50393235 & chr5;37290464**		**HR(95% CI)**	**p-value**
**Co-editing**			
	No	1(reference)	
	Yes	7.60(2.98-19.37)	**2.2e-5 *****
**Gender**			
Female		1(reference)	
Male		0.86(0.51-1.44)	0.56
**Ages**		1.00	**0.005****
**Stage**			
T1		1(reference)	
T2		1.14(0.59-2.20)	0.71
T3		1.67(0.89-3.15)	0.11
T4		4.93(1.87-13.03)	**0.001 ****
**Grade**			
Grade G1		1(reference)	
Grade G2		1.59(0.60-4.17)	0.35
Grade G3		2.35(0.87-6.32)	0.09
Grade G4		2.84(0.67-11.96)	0.16
**Fetoprotein**		1.00(1.00-1.00)	0.33

Note: *p < 0.05; **p < 0.01; ***p < 0.001

Supplementary data 1: The expression change of genes involved in 12,537 RNA co-edited pairs on the same chromosome.

Supplementary data 2: The expression changes of *ADAR1* and *ADAR2* between RNA co-edited samples and non-co-edited samples in HCC.

Supplementary data 3: The source data used to generate the main figures.
