# Supplementary material for: A-to-I RNA co-editing predicts clinical outcomes and is associated with immune cells infiltration in hepatocellular carcinoma
Source: Commun Biol. 2024 Jul 9;7:838. doi: 10.1038/s42003-024-06520-y (PMC11233613; doi:10.1038/s42003-024-06520-y)
Supplement: Supplementary file 3 — Description of Additional Supplementary Materials [file 42003_2024_6520_MOESM3_ESM.docx]

**Description of Additional Supplementary Files**

**File name:** Supplementary Data 1

**Description:** The expression change of genes involved in 12,537 RNA co-edited pairs on the same chromosome.

**File name:** Supplementary Data 2

**Description:** The expression changes of ADAR1 and ADAR2 between RNA co-edited samples and non-co-edited samples in HCC

**File name:** Supplementary Data 3

**Description:** The source data used to generate the main figures.
